# Comparison of pressure-controlled ventilation with volume-controlled ventilation during one-lung ventilation: a systematic review and meta-analysis

**DOI:** 10.1186/s12871-016-0238-6

**Published:** 2016-08-31

**Authors:** Kyu Nam Kim, Dong Won Kim, Mi Ae Jeong, Yeong Hun Sin, Soo Kyung Lee

**Affiliations:** Department of Anesthesiology and Pain Medicine, Hanyang University Hospital, 222, Wangsimni-ro, Seongdonggu, Seoul 133-792 Republic of Korea

**Keywords:** One-Lung Ventilation, Respiration, artificial

## Abstract

**Background:**

Not only arterial hypoxemia but acute lung injury also has become the major concerns of one-lung ventilation (OLV). The use of pressure-controlled ventilation (PCV) for OLV offers the potential advantages of lower airway pressure and intrapulmonary shunt, which result in a reduced risk of barotrauma and improved oxygenation, respectively.

**Methods:**

We searched Medline, Embase, the Cochrane central register of controlled trials and KoreaMedto find publications comparing the effects of PCV with those of volume-controlled ventilation (VCV) during intraoperative OLV in adults. A meta-analysis of randomized controlled trials was performed using the Cochrane Review Methods.

**Results:**

Six studies (259 participants) were included. The PaO_2_/FiO_2_ ratio in PCV was higher than in VCV [weighted mean difference (WMD) = 11.04 mmHg, 95 % confidence interval (CI) = 0.30 to 21.77, *P =* 0.04, I^2^ = 3 %] and peak inspiratory pressure was significantly lower in PCV (WMD = −4.91 cm H_2_O, 95 % CI = −7.30 to –2.53, *P <* 0.0001, *I* ^2^ = 91 %). No differences in PaCO_2_, tidal volume, heart rate and blood pressure were observed. There were also no differences incompliance, plateau and mean airway pressure.

**Conclusions:**

Our meta-analysis provided the evidence of improved oxygenation in PCV. However, it is difficult to draw any definitive conclusions due to the fact that the duration of ventilation in the studies reviewed was insufficient to reveal clinically relevant benefits or disadvantages of PCV. Significantly lower peak inspiratory pressure is the advantage of PCV.

**Electronic supplementary material:**

The online version of this article (doi:10.1186/s12871-016-0238-6) contains supplementary material, which is available to authorized users.

## Background

One-lung ventilation (OLV) is necessary to facilitate surgical access or to isolate a lung during thoracic surgery procedure. During OLV, the intentionally collapsed lung,which is continuously perfused but not ventilated, develops an intrapulmonary shunt leading to arterial hypoxemia [1, 2]. Although arterial hypoxemia is still a critical intraoperative problem, avoidance of lung injury has become the major concern in OLV [3, 4]. Elevated airway pressure associated with mechanical ventilation is the important risk factor for ventilator-induced lung injury [5, 6]. In addition, inflammatory reactions and tissue injuries associated with lung re-expansion cause the ventilator-induced lung injury after OLV [7–10].

The ventilator settings, including tidal volume and positive end-expiratory pressure (PEEP), for OLV were generally the same as for two-lung ventilation (TLV) previously [11]. However, the use of the conventional large tidal volume has been identified as a major risk factor for ventilator-induced lung injury [12, 13]. Therefore, the current trend is towards the use of low tidal volumes (4–6 ml/kg) instead of large tidal volumes (10–12 ml/kg) for OLV [14, 15]. The use of pressure-controlled ventilation (PCV) for OLV is seen as an alternative ventilator mode aimed at avoiding high airway pressure. When OLV is started, the entire tidal volume is delivered to just one lung, resulting in increased airway pressure in that lung. Because of the possibility of ventilator-induced lung injury when applying volume-controlled ventilation (VCV), a preference for applying PCV has developed [16].

During OLV, PCV offers the advantages of lower airway pressure and a lower intrapulmonary shunt leading to a reduced risk of ventilator-induced lung injury and improved oxygenation, respectively [17–19]. However, others have reported that PCV results in poorer oxygenation [20, 21] and the effect of PCV on oxygenation is one of the areasof greatest controversy. We hypothesized that PCV is associated with improved oxygenation and lower inspiratory pressure in comparison with VCV. Therefore, the authors have performed a systematic review and meta-analysis comparing the effect of PCV with that of VCV during intraoperative OLV in adults.

## Methods

We used a systematic approach to find publications comparing pressure-controlled ventilation with volume-controlled ventilation during OLV. This study is based on the Cochrane Review Methods [22].

### Data sources& literature sources

We searched Medline, Embase, the Cochrane central register of controlled trials and KoreaMed for eligible studies from inauguration to 22 July 2014, using a combination of controlled vocabulary (MeSH, Emtree) and free text terms. Main keywords were OLV, PCV and VCV. Search strategies were modified suitably for each database (Additional file 1). We manually searched the reference lists of the retrieved studies, ClinicalTrials.gov and the WHO ICTRP for additional unpublished/published studies.

### Study selection

All the studies selected were independently identified by two reviewers (KNK and MAJ) based on predefined selection criteria. We screened the titles and abstracts of the identified studies and then screened the full textsof the studies marked for inclusion. Disagreement in the primary study selection was arbitrated by the third reviewer (DWK). Studies were included in our meta-analysis if they fulfilled the following criteria: (1) Literature type: randomized controlled trials in all published international journals without limitation of language or nationality. (2) Subjects: adult patients undergoing elective surgery requiring OLV. (3) Interventions: studies comparing the effect of PCV with VCV during OLV. (3) Outcomes: the primary outcome was the PaO_2_/FiO_2_ ratio, and secondary outcomeswere peak, plateau, and mean inspiratory airway pressures and postoperative events. Other parameters of gas exchange, including PaO_2_, SaO_2_, PaCO_2_, alveolar-arterial oxygen difference, intrapulmonary shunt were also collected. The outcome variables are mean differences between the groups at the designated times.

### Data extraction

Two reviewers (KNK and MAJ) independently extracted the data using a pre-specified data extraction form. The data extracted from the selected studies was confirmed by the third reviewer (DWK).

The following variables were extracted: (1) means and standard deviations of the outcome data in the PCV and VCV groups; (2) number of patients, type of surgery, and recruitment procedure for each group; (3) the protocol for using fraction of inspired oxygen during surgery, and ventilator settings such as tidal volume, respiratory rate, inspiratory/expiratory ratio, inspiratory pause and PEEP for each group; (4) the timepoint of measurement of outcome data and (5) the method of assessment. If the above variables were not mentioned in a study, we asked for the data via email.

### Assessment of methodological quality

The reviewer (KNK and MAJ) independently assessed the risk of bias in each study using the Cochrane risk of bias tool. This tool assesses randomized controlled trials by evaluating the reported methods for random sequence generation, concealment of allocations, blinding of participants, personnel and the outcome assessor, incompleteness of outcome data, selective outcome reporting, and other possible sources of risk of bias. Discrepancies between the two reviewers were resolved face-to-face.

### Statistical analysis

The continuous variables such as PaO_2_/FiO_2_ ratio, intrapulmonary shunt and airway pressure were obtained at designated times. We analyzed the continuous data using weighted mean differences (WMD) employing the generic inverse variance method, and reported mean differences and their associated 95 % confidence intervals (CI). Heterogeneity between studies was assessed using the χ2 test and the I^2^ statistic [23]. We considered that an I^2^ statistic >50 % and aχ2 test with a *P* value <0.10 indicated statistical heterogeneity. We used random-effects models if clinical heterogeneity or statistical heterogeneity were detected. A subgroup analysis was performed to eliminate the effect of paravertebral block which has an effect of sympathetic nervous system blockade that inhibits hypoxic pulmonary vasoconstriction (HPV). A subgroup analysis between low tidal volume (6–8 ml/kg) and high tidal volume (9–10 ml/kg) was also performed because the reduction of tidal volume to 6–8 ml/kg was beneficial in terms of occurrence of respiratory complications and the length of hospital stay [24].

In meta-analyses that include the results of cross-over studies (in which patients cross over from one treatment to another during the course of the trial), there is a risk of bias due toa carry-over effects [25]. We conducted a sensitivity analysis in situations where this could affect our estimates.

All statistical analyses were conducted using RevMan version 5.2. When the number of studies included was less than 10, we did not evaluate publication bias because of the low statistical power.

## Results

### Identification of studies

**Fig. 1:**
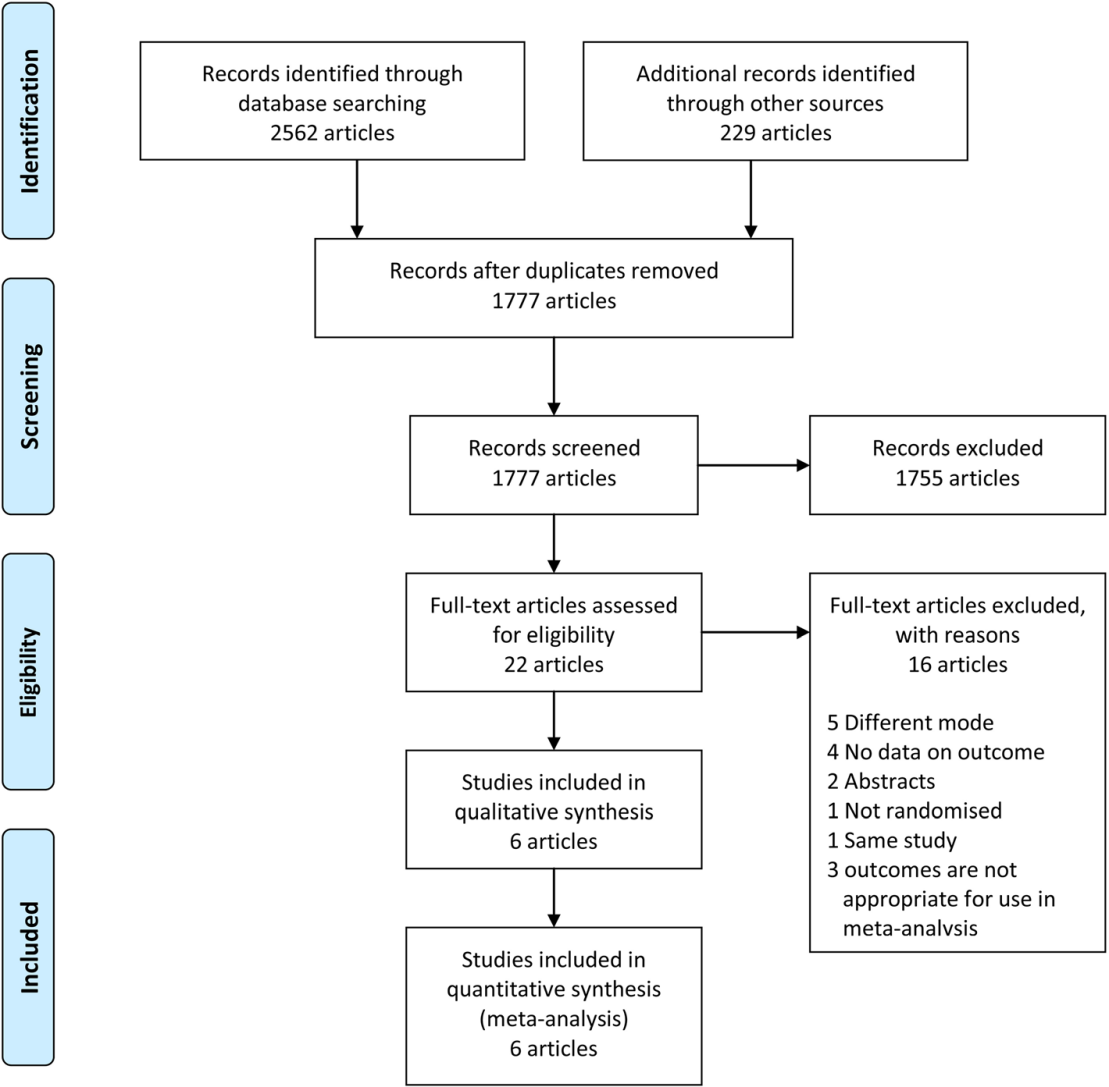
Flow-chart of the literature search strategy

Initial searches of the databases yielded 2791 articles. After removing 1014 duplicated articles, 1755 further publications were eliminated as it wasclear from their titles and abstracts that they did not fulfill the selection criteria. For the remaining 22 articles, we obtained full manuscripts, and, following scrutiny of these, identified six articles describing potentially relevant studies; the 16 others were excluded because of use of a different mode of ventilation (five articles), two abstracts, no available outcome data (four articles), study design not randomized (one article), andthe same study data reported twice (one article). One article was excluded because of thoracotomies for robotic-assisted esophagectomy with prone position and twoarticles were excluded to remove variables that might affect oxygenation because cardiopulmonary bypass influences HPV and oxygenation. Hence, six studies [18, 21, 26–29] and 259 participants were included in this review (Fig. 1).

### Study characteristics and patient populations

The included articles were published in four countries: South America, Saudi Arabia, Spain (2), and Turkey (2) between 1997 and 2014. The patients in six studies [18, 21, 26–29] underwent thoracotomies for lung operations such as pneumectomy, lobectomy and wedge resection. The operating position in all cases was lateral decubitus. Each study used the same FiO_2_ and ventilator settings throughout each OLV. Five were crossover studies [18, 21, 27–29] which applied PCV for 30 min followed by VCV, in one experimental group, and the reverse order in the other. The patients were allocated randomly to one of the two groups and all measurements were made 30 min after starting each ventilation mode. One study [26] was non-crossover study (Table 1).

**Table 1:** The characteristics of the included randomized controlled trials comparing pressure-controlled ventilation with volume-controlled ventilation

					Ventilator settings									
Study and year	ASA	Patients	(n)	Surgery	Tidal Volume			Target CO_2_	I:E ratio	Inspiratory pause	FiO_2_	PEEP (cmH_2_O)	RM	Timing of RM
					PCV	VCV	Kg							
Al Shehri [29] 2014	II-III	PCV-VCV	14	Thoracotomy for lung disease	6 ml/kg	6 ml/kg	PBW	PaCO_2_35-45 mmHg	1:2.5	N/D	0.5	5	Yes	At every 30 min
		VCV-PCV	14											
ErenOngur [27] 2010	I-II	PCV-VCV	15	Thoracotomy for lung disease	6–7 ml/kg	6 –7 ml/kg	TBW	PaCO_2_35 –45 mmHg	N/D	N/D	0.5	0	Yes	Before returning to TLV
		VCV-PCV	15											
Montes [21] 2010	I-III	PCV-VCV	21	Thoracotomy for lung disease	6 ml/kg	6 ml/kg	TBW	ETCO_2_25 –30 mmHg	1:3	10%	1.0	5	No	
		VCV-PCV	20											
Pardos [26] 2009	N/D	PCV	55	Thoracotomy for lung disease	8 ml/kg	8 ml/kg	TBW	PaCO_2_35 –40 mmHg	1:2	15%	1.0	5	Yes	At 20 min after OLV
		VCV	55											
Tugrul M [18] 1997	I-III	PCV-VCV	24	Thoracotomy for lung disease	10 ml/kg	10 ml/kg	TBW	PaCO_2_34 –45 mmHg	1:3	10%	1.0	0	No	
		VCV-PCV	24											
Unzueta MC [28] 2007	II-III	PCV-VCV	29	Thoracotomy for lung disease	9 ml/kg	9 ml/kg	TBW	ETCO_2_30 –35 mmHg	1:2	0.9 s	1.0	0	No	
		VCV-PCV	28											

The respiratory rate was adjusted to maintain PaCO_2_ or ETCO_2_

*ASA* American Society of Anesthesiologists’ classification, (*n*) number of cases, *PCV* pressure-controlled ventilation, *VCV*, volume-controlled ventilation, *I:E ratio* the inspiratory to expiratory time ratio, *FiO*_*2*_ fraction of inspired oxygen, *PEEP* positive end-expiratory pressure, *RM* recruitment maneuver, *PBW* predicted body weight, *PaCO*_*2*_ partial pressure of carbon dioxide, *ETCO*_*2*_ end-tidal carbon dioxide, *TLV* two-lung ventilation, *OLV* one-lung ventilati on, *N/D* no data

### Quality of the included studies

**Fig. 2:**
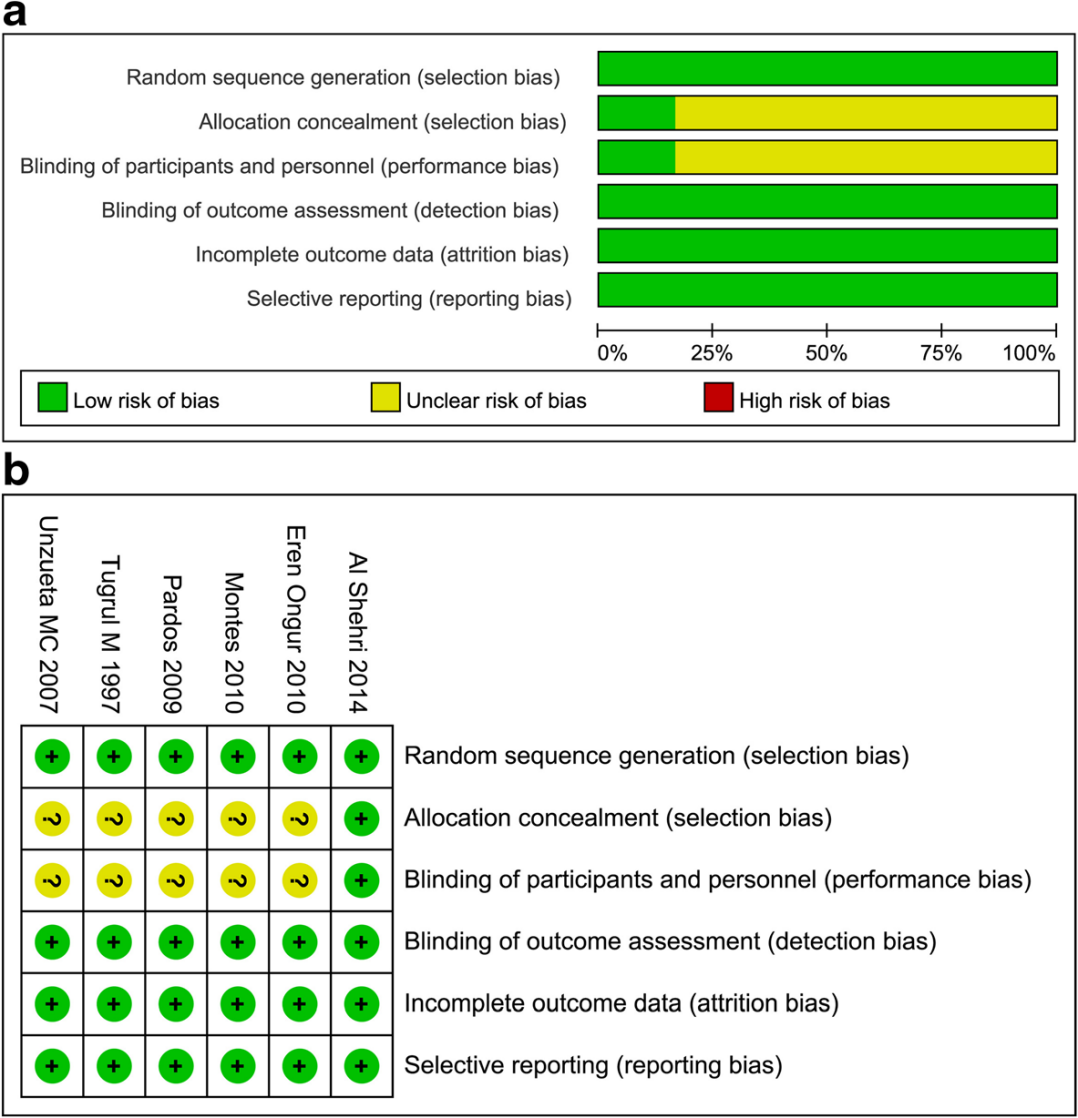
**a** Risk-of-bias graph of all the included randomized controlled trials. **b** Risk-of-bias summary of all the included randomized controlled trials

All the studies used a random allocation method and one study [29] described the allocation concealment and blinding methods in detail. Although the risk of selective reporting and incomplete outcome data was low, the risk of allocation concealment and blinding was unclear in most studies. Risk-of-bias graphs and summaries are presented in Fig. 2a and b.

### Statistical heterogeneity

PaO_2_/FiO_2_ ratio, PaCO_2_, tidal volume, heart rate and blood pressure showed no significant heterogeneity among the studies (I^2^ statistic value < 40 %). Those of peak, plateau, and mean inspiratory airway pressure and compliance displayed heterogeneity (I^2^ statistic value > 60 %).

### Gas exchange

**Fig. 3:**
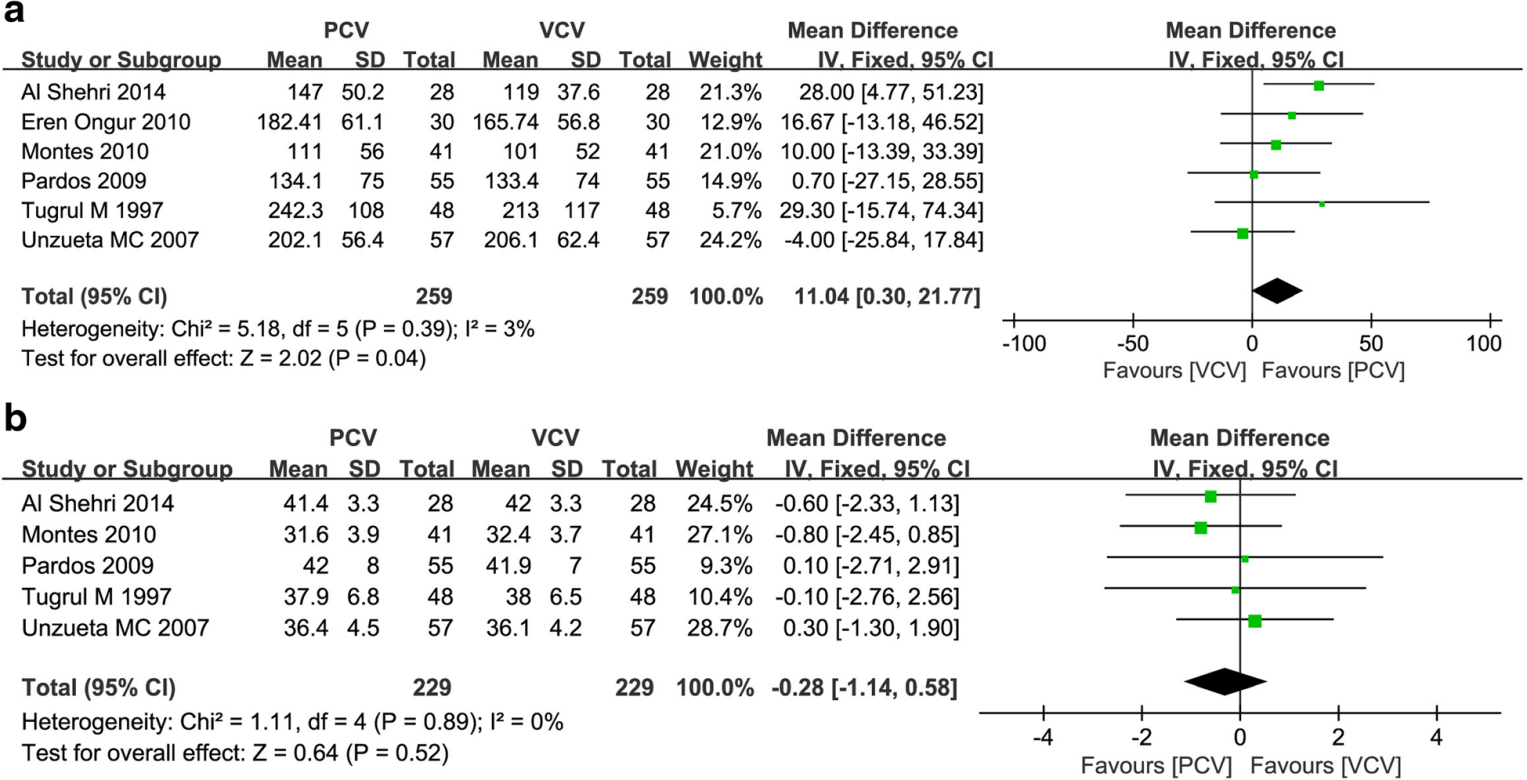
Meta-analysis of the effect of intraoperative ventilation with pressure-controlled ventilation compared with volume-controlled ventilation. **a** Impact on PaO_2_/FiO_2_ ratio (mmHg). **b** Impact on PaCO_2_ (mmHg)

The PaO_2_/FiO_2_ ratio was extracted from 6 randomized trials [18, 21, 26–29]. We found a higher PaO_2_/FiO_2_ ratio in PCV than in VCV (WMD = 11.04 mmHg, 95 % CI = 0.30 to 21.77, *P =* 0.04) (Fig. 3a), but they did not differ in PaCO_2_ (WMD = −0.28 mmHg, 95 % CI = −1.14 to 0.58, *P =* 0.52) (Fig. 3b).

### Airway pressure and compliance

**Fig. 4:**
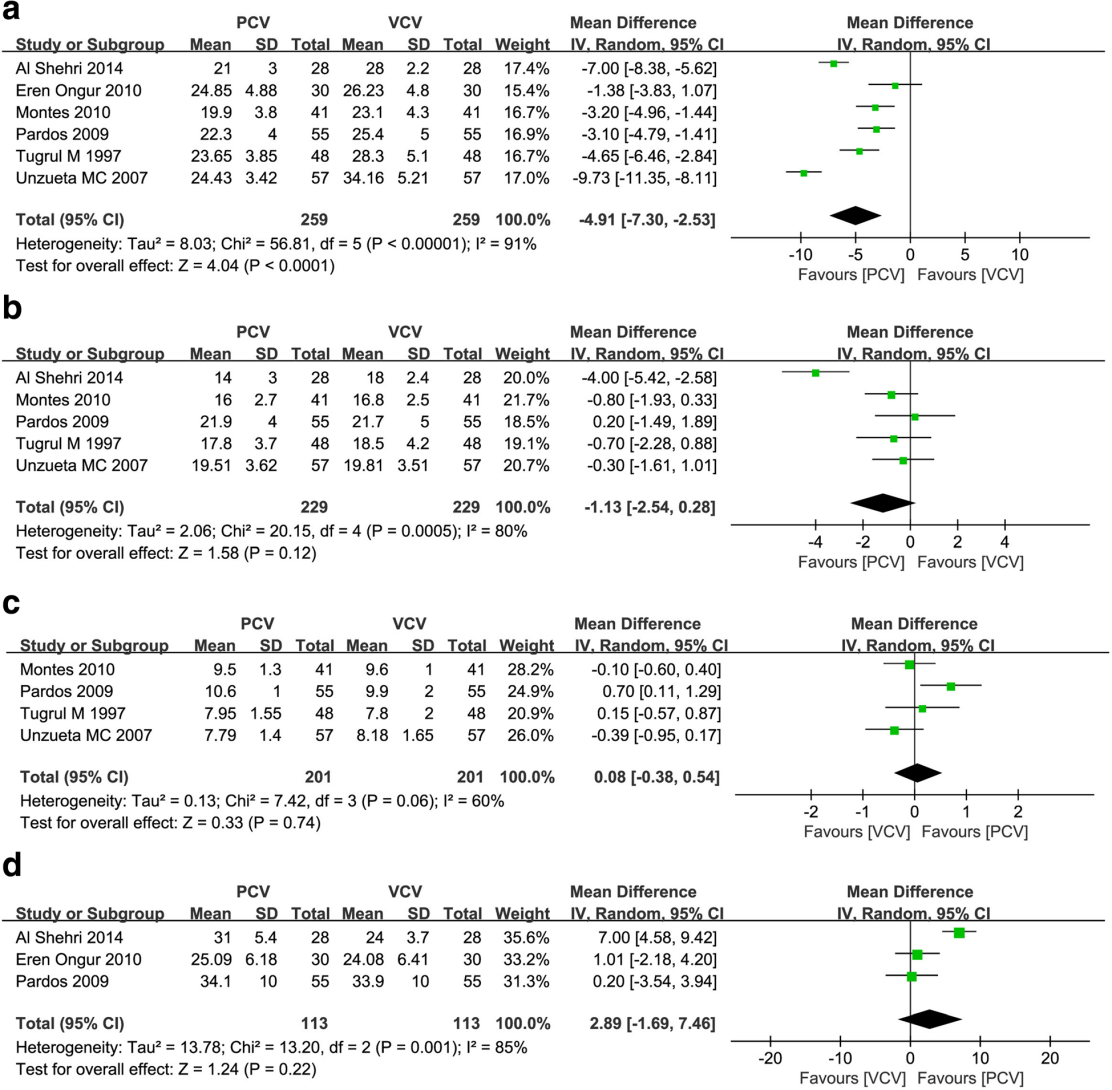
Meta-analysis of the effect of intraoperative ventilation with pressure-controlled ventilation compared to volume-controlled ventilation. **a** Impact on peak airway pressure (cmH_2_O). **b** Impact on plateau airway pressure (cmH_2_O). **c** Impact on mean airway pressure (cmH_2_O). **d** Impact on compliance (ml/cmH_2_O)

Peak inspiratory pressure was significantly lower in PCV (WMD = −4.91 cmH_2_O, 95 % CI = −7.30 to –2.53, *P <* 0.0001) (Fig. 4a). However, there were no differences in plateau (WMD = −1.13 cmH_2_O, 95 % CI = −2.54 to 0.28, *P =* 0.12) (Fig. 4b), mean airway pressure (WMD = 0.08 cmH_2_O, 95 % CI = −0.38 to 0.54, *P =* 0.74) (Fig. 4c) or compliance (WMD = 2.89 ml/cmH_2_O, 95 % CI = −1.69 to 7.46, *P =* 0.22) (Fig. 4d).

### Tidal volume and hemodynamic variables

Tidal volume which was measured in each ventilator mode was reported in 3studies [18, 21, 28]. There was no difference in tidal volume between PCV and VCV (WMD = 0.83 ml, 95 % CI = −21.89 to 21.59, *P =* 0.99) during OLV. There were also no differences in heart rate (WMD = −0.70 beat/min, 95 % CI = −3.47 to 2.07, *P =* 0.62) or blood pressure (WMD = −0.43 mmHg, 95 % CI = −3.94 to 3.09, *P =* 0.81).

### Postoperative events

Postoperative events were reported in two studies [26, 29]. The length of postoperative stay and development of lung injury/adult respiratory distress syndrome (ARDS) were not different between groups. Mortality within 30 days was not observed in both groups.

### Subgroup analysis

**Fig. 5:**
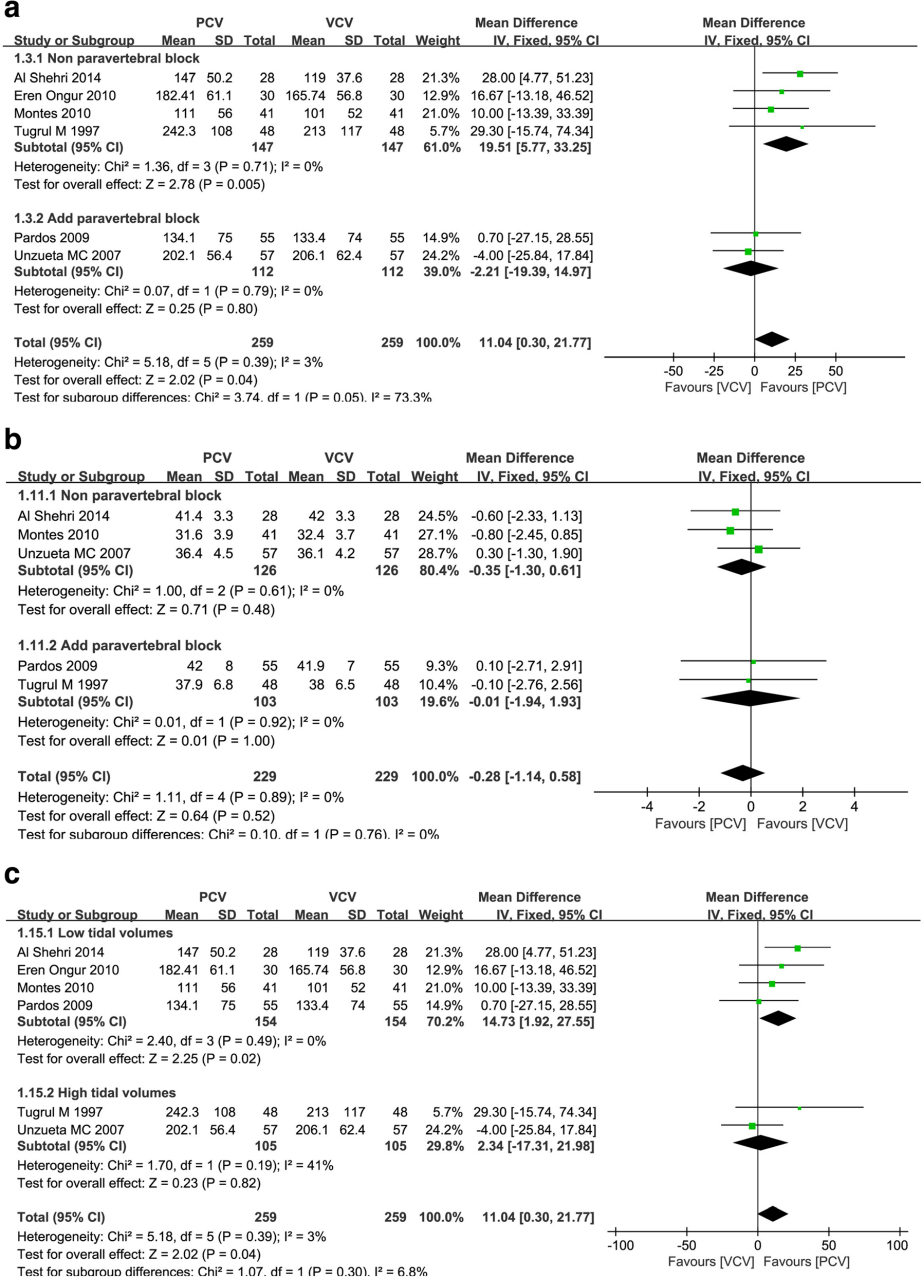
Subgroup analysis during pressure-controlled ventilation compared to volume-controlled ventilation. **a** The effect of paravertebral block on PaO_2_/FiO_2_ ratio (mmHg). **b** The effect of paravertebral block on PaCO_2_ (mmHg). **c** The effect of tidal volume (6–8 ml/kg vs 9–10 ml/kg) on PaO_2_/FiO_2_ ratio

A subgroup analysis including the studies in which paravertebral blockwas not performed showed that PCV was more effective than VCV with respect to the PaO_2_/FiO_2_ratio (WMD = 19.51 mmHg, 95 % CI = 5.77 to 33.25, *P =* 0.005) (Fig. 5a). However, paravertebral block with PCV showed no difference in PaCO_2_(WMD = −0.35 mmHg, 95 % CI = −1.30 to 0.61, *P =* 0.48) (Fig. 5b). The studies which applied low tidal volume (6–8 ml/kg) with PCV had the higher PaO_2_/FiO_2_ ratio than low tidal volume (6–8 ml/kg) with VCV in our subgroup analysis (WMD = 14.73 mmHg, 95 % CI = 1.92 to 27.55, *P =* 0.02) (Fig. 5c).

### Sensitivity analysis

We performeda sensitivity analysis to evaluate the influence of the crossover study design. The results for 5 crossover studies [18, 21, 27–29] showed an increased PaO_2_/FiO_2_ ratio in PCV (WMD = 12.84 mmHg, 95 % CI = 1.21 to 24.47, *P =* 0.03). A minor increase in mean airway pressure in PCV (WMD = 0.70 mmHg, 95 % CI = 0.11 to 1.29, *P =* 0.02) was detected in the analysis of one non-crossover study [26]. The sensitivity analysis of the crossover study design did not affect the resultsfor PaCO_2_ and peak and plateau airway pressure (Table 2).

**Table 2:** Sensitivity analysis of crossover study and abstract on the meta-analysis

Outcome		Studies (n)	Patients (n)	WMD	95 % CI	I^2^ (%)	*P* value
PaO_2_/FiO_2_ ratio	Total	6 [18, 21, 26–29]	259	11.04 mmHg	0.30 to 21.77	3	0.04
	Crossover studies	5 [18, 21, 27–29]	204	12.84 mmHg	1.21 to 24.47	12	0.03
	Non-crossover studies	1 [26]	55	0.70 mmHg	−27.15 to 28.55	0	0.96
PaCO_2_	Total	5 [18, 21, 26, 28, 29]	229	−0.28 mmHg	−1.14 to 0.58	0	0.52
	Crossover studies	4 [18, 21, 28, 29]	174	−0.32 mmHg	−1.22 to 0.58	0	0.49
	Non-crossover studies	1 [26]	55	0.10 mmHg	−2.71 to 2.91	65	0.94
Peak inspiratory Pressure	Total	6 [18, 21, 26–29]	259	−4.91cmH_2_O	−7.30 to −2.53	91	<0.0001
	Crossover studies	5 [18, 21, 27–29]	204	−5.27cmH_2_O	−7.98 to −2.57	92	<0.0001
	Non-crossover studies	1 [26]	55	−3.10cmH_2_O	−4.79 to −1.41	0	<0.0001
Plateau inspiratory Pressure	Total	5 [18, 21, 26, 28, 29]	229	−1.13cmH_2_O	−2.54 to 0.28	80	0.12
	Crossover studies	4 [18, 21, 28, 29]	174	−1.44cmH_2_O	−3.06 to 0.18	83	0.08
	Non-crossover studies	1 [26]	55	0.20cmH_2_O	−1.49 to 1.89	0	0.82
Mean inspiratory Pressure	Total	4 [18, 21, 26, 28]	201	0.08cmH_2_O	−0.38 to 0.54	60	0.74
	Crossover studies	3 [18, 21, 28]	146	−0.15cmH_2_O	−0.48 to 0.18	0	0.38
	Non-crossover studies	1 [26]	55	0.70cmH_2_O	0.11 to 1.29	0	0.02

(*n*) the number of cases, *WMD* weighted mean difference, *CI* confidence interval, *PCV* pressure-controlled ventilation, *VCV* volume-controlled ventilation, *PaO*_*2*_ partial pressure of oxygen, *FiO*_*2*_ fraction of inspired oxygen, *PaCO*_*2*_ partial pressure of carbon dioxide

## Discussion

Many studies have been performed in animals and humansto discover the most effective and safe ventilator strategies for OLV, and PCV has been recognized as a suitable method. PCV generates a square pressure waveform (constant inspired flow), which is results by the high delivering flow into the ventilator circuit. Theoretically, a decelerating inspiratory flow pattern results in a more even distribution of tidal volume, facilitating recruitment of insufficiently ventilated lung units and improvingoxygenation [30–33]. In addition, the rapid alveolar inflation caused by the high initial flow rate in PCV avoids regional overdistension because of the homogeneous gas distribution, and enables better ventilation perfusion matching [34]. Although the difference is minimal, PCV is more effective than VCV in oxygenation and significantly lower peak inspiratory pressure in PCV was observed in this meta-analysis.

Despite the aforementioned benefits of PCV, the magnitude of the increase in PaO_2_/FiO_2_ ratio in PCV was quite limited. There are several explanations for this result. First, PCV tends to produce a higher mean airway and alveolar pressure than VCV because of the rapid delivery of most of the tidal volume in the early part of inspiration [32], and in passive inflation conditions mean airway pressure closely correlates with alveolar ventilation, arterial oxygenation and cardiovascular function [35]. As a result, the increased mean airway pressure in PCV is associated with improved oxygenation. However,the fact that there was no difference in mean airway pressure between the two groups in this meta-analysis demonstrates the weakness of the effect of PCV on oxygenation during OLV. In the absence of recruitment, compression of the intra-alveolar vessels due to the high mean airway pressure during inflation rather increases the intrapulmonary shunt [32].

Other reason could be the lack of PEEP and the use of a recruitment maneuver which was applied only in three studies [21, 26, 29] and 2 studies [26, 29], respectively. PCV with PEEP is associated with an improvement in oxygenation and providing lower airway pressure during OLV [17]. In addition, alveolar recruitment strategies decrease alveolar dead space and improve gas exchange during OLV [17, 36, 37]. These effects of PEEP and recruitment maneuvers during OLV may encourage the recruitment of insufficiently ventilated lungs,and this could be facilitated by the decelerating inspiratory flow pattern of PCV.

Lastly, PCV per se may have no clinical benefits in terms of improving oxygenation. In studies of ARDS, there was no significant effect of PCV on gas exchange [34, 38, 39]. The results of one experimental study even suggest that the high initial flow rates of PCV cause lung tissue injury and reduce gas exchange rather than protecting the lung [40].

Another interesting issue raised by this meta-analysis is how HPV influences the effect of PCV on oxygenation. The subgroup analysis performed to eliminate the effect of paravertebral block showed that the PaO_2_/FiO_2_ ratiowas better in PCV than VCV. The intrapulmonary shunt occurring during OLV could be compensated for by the blood flow diverted to the ventilated lung by HPV [41]. Paravertebral block with local anesthetics has an effect of sympathetic nervous system blockade that inhibits HPV and thereby produces a larger shunt and a decrease in oxygenation during OLV [42–44]. Based on these results, it appears that the larger shunt due to the inhibition of HPV reduces the favorable effects of PCV in improving oxygenation.

Although we detected a significantly reduced peak inspiratory pressure in PCV in our meta-analysis, this difference in peak inspiratory pressure did not constitute a specific advantage of PCV, according to our meta-analysis. Since the airway pressure caused by resistance factors is dependent on flow during ventilatory delivery of tidal volume, airway pressure and alveolar pressure are identical only when there is no flow [45]. Thus, peak inspiratory pressure which was measured while high inspiratory gas flow occurred could not reflect alveolar pressure precisely. Furthermore, the incidence of barotrauma is strongly correlated with plateau airway pressure rather than peak inspiratory pressure, and to avoid overinflation of the ventilated lung during OLV, < 35 cmH_2_O of peak inspiratory pressure and < 25 cmH_2_O of plateau airway pressure are recommended [46, 47]. Plateau airway pressure of < 30 cmH_2_O probably does not deteriorate the development of lung injury/ARDS after OLV [4, 15]. Because both the peak and plateau pressures werebelow these limits in all the studies included in this meta-analysis, and no difference in plateau airway pressure was detected between the two groups, no clinical merit of PCV was found despite the lower peak inspiratory pressure in PCV.

In addition, as we mentioned before, the focus of mechanical ventilation has moved to prevent the development of lung injury. However, few studies have been performed to evaluate postoperative lung complications after OLV using different ventilator modes. Although we found no differences in the length of postoperative stay, development of lung injury/ARDS and mortality within 30 days between the two groups, it is not reasonable to draw any conclusions about the effectiveness of different ventilator modes from only two studies.

In the ways that both OLV and ARDS are ventilated with a small lung volume,the so-called baby lung in ARDS and one lung in OLV, OLV is similar to ARDS [3]. Hence, reducing the tidal volume, which is a protective ventilation strategy in ARDS,may also be protective in the case of OLV. Michelet et al. reported that a protective ventilation strategy group, ventilated with a tidal volume of 5 ml/kg and PEEP of 5 cmH_2_O during OLV developed lower proinflammatory systemic responses (IL-1β,IL-6 and IL-8), and had improved lung function and earlier extubation [48]. Licker et al., have reported clinical benefits of low tidal volumefrom the secondary analysis of an observational cohort [49]. The use of a low tidal volume with PEEP and recruitment maneuvers during OLV significantly reduced the incidence of lung injury and atelectasis after lung cancer resection. Our subgroup analysis also revealed that the studies which applied low tidal volume (6–8 ml/kg) with PCV had the higher PaO_2_/FiO_2_ ratio than low tidal volume (6–8 ml/kg) with VCV (Fig. 5c). Nevertheless, only few studies which have been reported were using low tidal volume of 6–8 ml/kg for OLV. Because the adverse effects of ventilation with a high tidal volume can exacerbate lung injury, the beneficial effects of PCV may be too weak to be detected. Therefore, well-controlled randomized studies using a low tidal volume in each ventilator mode during OLV are needed to accurately assess differences between PCV and VCV.

### Limitations of our study

Our meta-analysis has several limitations. First of all, the relatively small number of patients was included in this study. Intervention effects can be significantly over stated in small trials with incomplete allocation sequence generation, allocation concealment, and double blinding [50]. However the patients in all the studies in the meta-analysis had been randomly allocated and the outcome data for mechanical ventilation involved objective measurements. Consequently, we believe that the risk of bias in these studies was low and the intervention effects were properly estimated.

Secondly, most of the studies included in it are crossover studies, and it could be agreed that the analysis has a problem with reliability. Crossover studies have the problem of carryover effects that may affect the analysis of interventions. However, PaO_2_ was maintained after 20 min of ventilation [51] and ventilation for more than 20 min can washout the influence of previous ventilator settings. Therefore the fact that all the outcome data of our crossover studies were obtained after 30 min of ventilation may have eliminated any carryover effects. Furthermore, the crossover study design has the advantage of reducing the effect of individual differences in terms of age, pulmonary function and severity of disease, which can lead to misinterpretation of the results. According to our sensitivity analysis of the crossover studies, gas exchange was more efficient in PCV (Table 2). This may have been due toan underestimation of the effect of PCV in the non-crossover studies due to individual factors. Nevertheless, owing to the slight effect on gas exchange of PCV in comparison with VCV, the clinical significance of this difference is small. Furthermore, there still remains the problem that the 30 min of temporary ventilation was not sufficient to reveal clinically-relevant benefits or adverse effects of PCV.

Lastly, evaluating the postoperative pulmonary complications after OLV using different ventilator modes is important to assess whether PCV could lower the theoretical risk of ventilator-induced lung injury. However, few studies have been performed to evaluate postoperative lung complications after OLV.

## Conclusions

In conclusion, our meta-analysis has provided evidence that peak inspiratory pressure is significantly lower in PCV. In terms of oxygenation, although the use of low tidal volume with PCV was associated with improved oxygenation, no definitive conclusions could be drawn because the duration of ventilation in the studies reviewed was insufficient to rule out important differences if they exist. Most of the studies included in the meta-analysis were crossover studies involving 30 min of ventilation, and used high tidal volumes that are risk factors for lung injury. Therefore, to evaluate the influence of PCV on occurrence of the lung injury/ARDS, well-controlled randomized non-crossover studies using low tidal volumes with adequate durations of each ventilator mode, are needed.

## Additional file

Additional file 1: Table S1. Search strategies of each database. (DOCX 23 kb)

## Abbreviations

ARDS: Adult respiratory distress syndrome
CI: Confidence intervals
HPV: Hypoxic pulmonary vasoconstriction
OLV: One-lung ventilation
PCV: Pressure-controlled ventilation
PEEP: Positive end-expiratory pressure
TLV: Two-lung ventilation
VCV: Volume-controlled ventilation
WMD: Weighted mean difference
