# Supplementary material for: Comparison of pressure-controlled ventilation with volume-controlled ventilation during one-lung ventilation: a systematic review and meta-analysis
Source: BMC Anesthesiol. 2016 Aug 31;16:72. doi: 10.1186/s12871-016-0238-6 (PMC5007729; doi:10.1186/s12871-016-0238-6)
Supplement: Additional file 1: Table S1. — Search strategies of each database. (DOCX 23 kb) [file 12871_2016_238_MOESM1_ESM.docx]

**Supplemental Table 1** Search strategies of each database.

| **MEDLINE** | |
| --- | --- |
| 1. | ventilate[tiab] OR ventilate'[tiab] OR ventilated[tiab] OR ventilated'[tiab] OR ventilateddependent[tiab] OR ventilatilation[tiab] OR ventilation'[tiab] OR ventilation's[tiab] OR ventilational[tiab] ventilations[tiab] ventilative[tiab] OR ventilator[tiab] OR ventilator'[tiab] OR ventilator's[tiab] OR ventilators'[tiab] ventilatyro[tiab] OR Ventilation[tiab] |
| 2. | ("Respiration, Artificial"[Mesh:NoExp]) OR "Positive-Pressure Respiration"[Mesh:NoExp] |
| 3. | 1 OR 2 |
| 4. | "Thoracic Surgery"[tiab] OR "Thoracic Surgical"[tiab] OR "Pulmonary Surgical"[tiab] OR "Pulmonary Surgery"[tiab] OR "one-lungventilation"[tiab] OR "One Lung Ventilation"[tiab] OR "Single-Lung Ventilation"[tiab] OR "Single Lung Ventilation"[tiab] OR "Single-Lung Ventilations"[tiab] OR "Lung Separation Techniques"[tiab] OR "Lung Separation Technique"[tiab] OR "Bronchoscopies"[tiab] OR "Bronchoscopy"[tiab] OR "Bronchoscopic"[tiab] OR "Collapse Therapy"[tiab] OR "Collapse Therapies"[tiab] OR "Pneumothorax"[tiab] OR "Pneumothoraxs"[tiab] OR "Pneumonectomies"[tiab] OR "Lung Volume Reduction"[tiab] OR "Lung Volume Reductions"[tiab] OR "Pneumonectomy"[tiab] |
| 5. | (("One-Lung Ventilation"[Mesh]) OR "Thoracic Surgical Procedures"[Mesh:NoExp]) OR "Pulmonary Surgical Procedures"[Mesh] |
| 6. | (("Thoracotomy"[Mesh]) OR "Thoracostomy"[Mesh]) OR "Thoracoscopy"[Mesh] |
| 7. | "thorax surgery"[tiab] OR "thorax surgeries"[tiab] OR thoracotomy[tiab] |
| 8. | 4 OR 5 OR 6 OR 7 |
| 9. | 8 AND 3 |
| 10. | 7 AND (("randomized controlled trial"[Publication Type] OR "controlled clinical trial"[Publication Type] OR randomized[tiab] OR placebo[tiab] OR "clinical trials as topic"[Mesh:noexp] OR randomly[tiab] OR trial[ti])) NOT ((animals[Mesh] NOT (humans[Mesh] AND animals[Mesh]))) |
| 11. | 8 NOT ("review"[Publication Type] OR "review literature as topic"[MeSH Terms]) |
| **EMBASE** | |
| 1. | ventilate:ab,ti OR ventilated:ab,ti OR ventilateddependent:ab,ti OR ventilatilation:ab,ti OR ventilational:ab,ti OR ventilations:ab,ti OR ventilative:ab,ti OR ventilator:ab,ti OR ventilators:ab,ti OR ventilatyro:ab,ti OR Ventilation:ab,ti |
| 2. | ' artificial ventilation'/de OR 'lung ventilation'/exp |
| 3. | 1 OR 2 |
| 4. | 'one lung ventilation'/exp OR 'thorax surgery'/de OR 'esophagus surgery'/exp OR 'lung surgery'/exp OR 'thoracotomy'/exp OR 'video assisted thoracoscopic surgery'/exp |
| 5. | 'thoracic surgery':ab,ti OR 'thoracic surgical':ab,ti OR 'pulmonary surgical':ab,ti OR 'pulmonary surgery':ab,ti OR 'one-lung ventilation':ab,ti OR 'one lung ventilation':ab,ti OR 'single-lung ventilation':ab,ti OR 'single lung ventilation':ab,ti OR 'single-lung ventilations':ab,ti OR 'lung separation techniques':ab,ti OR 'lung separation technique':ab,ti OR 'bronchoscopies':ab,ti OR 'bronchoscopy':ab,ti OR 'bronchoscopic':ab,ti OR 'collapse therapy':ab,ti OR 'collapse therapies':ab,ti OR 'pneumothorax':ab,ti OR 'pneumothoraxs':ab,ti OR 'pneumonectomies':ab,ti OR 'lung volume reduction':ab,ti OR 'lung volume reductions':ab,ti OR 'pneumonectomy':ab,ti OR 'thorax surgery':ab,ti OR 'thorax surgeries':ab,ti OR thoracotomy:ab,ti |
| 6. | 4 OR 5 |
| 7. | 6 AND 3 |
| 8. | 7 AND 'crossover procedure'/exp OR 'double blind procedure'/exp OR 'randomized controlled trial'/exp OR 'single blind procedure'/exp OR random* OR factorial* OR crossover* OR 'cross over' OR 'cross-over' OR placebo* OR (doubl* AND blind*) OR (singl* AND blind*) OR assign* OR allocat* OR volunteer* |
| 9. | 8 NOT ('letter'/it OR 'review'/it) |
| 10. | 9 NOT ('animal experiment'/de OR 'animal model'/de OR 'animal tissue'/de) |
| **COCHRANE** | |
| 1. | ventilate or ventilate' or ventilated or ventilated' or ventilated dependent or ventilatilation or ventilation' or ventilation's or ventilational ventilations ventilative or ventilator or ventilator' or ventilator's or ventilators' ventilatyro or Ventilation:ti,ab,kw (Word variations have been searched) |
| 2. | MeSH descriptor: [Respiration, Artificial] this term only |
| 3. | MeSH descriptor: [Positive-Pressure Respiration] this term only |
| 4. | #1-3/or |
| 5. | "Thoracic Surgery" or "Thoracic Surgical" or "Pulmonary Surgical" or "Pulmonary Surgery" or "one-lung ventilation" or "One Lung Ventilation" or "Single-Lung Ventilation" or "Single Lung Ventilation" or "Single-Lung Ventilations" or "Lung Separation Techniques" or "Lung Separation Technique" or "Bronchoscopies" or "Bronchoscopy" or "Bronchoscopic" or "Collapse Therapy" or "Collapse Therapies" or "Pneumothorax" or "Pneumothoraxs" or "Pneumonectomies" or "Lung Volume Reduction" or "Lung Volume Reductions" or "Pneumonectomy":ti,ab,kw (Word variations have been searched) |
| 6. | MeSH descriptor: [One-Lung Ventilation] explode all trees |
| 7. | MeSH descriptor: [Thoracic Surgical Procedures] this term only |
| 8. | MeSH descriptor: [Pulmonary Surgical Procedures] explode all trees |
| 9. | MeSH descriptor: [Thoracotomy] explode all trees |
| 10. | MeSH descriptor: [Thoracostomy] explode all trees |
| 11. | MeSH descriptor: [Thoracoscopy] explode all trees |
| 12. | "thorax surgery" or "thorax surgeries" or thoracotomy:ti,ab,kw (Word variations have been searched) |
| 13. | #5-12/or |
| 14. | #4 and #13 |
| 15. | #14/Trials |
| **KOREAMED** | |
| 1. | ventilation[ALL] OR ventilated[ALL] OR ventilator[ALL] OR ventilated dependent[ALL] OR ventilational[ALL] OR ventilative[ALL] OR ventilatyro[ALL] |
| 2. | "Thoracic Surgery"[ALL] OR "Pulmonary Surgery"[ALL] OR "One Lung Ventilation"[ALL] OR "Single Lung Ventilation"[ALL] OR "Lung Separation Techniques"[ALL] OR "Lung Separation Technique"[ALL] OR "Bronchoscopy"[ALL] OR "Collapse Therapy"[ALL] OR "Collapse Therapies"[ALL] OR "Pneumothorax"[ALL] OR "Pneumothoraxs"[ALL] OR "Lung Volume Reduction"[ALL] OR "Pneumonectomy"[ALL] OR Thoracotomy[ALL] |
| 3. | 1 AND 2 |
